# Invasive Shrub Mapping in an Urban Environment from Hyperspectral and LiDAR-Derived Attributes

**DOI:** 10.3389/fpls.2016.01528

**Published:** 2016-10-21

**Authors:** Curtis M. Chance, Nicholas C. Coops, Andrew A. Plowright, Thoreau R. Tooke, Andreas Christen, Neal Aven

**Affiliations:** ^1^Department of Forest Resources Management, Faculty of Forestry, University of British ColumbiaVancouver, BC, Canada; ^2^City of SurreySurrey, BC, Canada; ^3^Department of Geography, Faculty of Arts, University of British ColumbiaVancouver, BC, Canada

**Keywords:** invasive species, hyperspectral imaging, imaging spectroscopy, LiDAR, *Rubus armeniacus*, *Hedera helix*, random forests, urban environments

## Abstract

Proactive management of invasive species in urban areas is critical to restricting their overall distribution. The objective of this work is to determine whether advanced remote sensing technologies can help to detect invasions effectively and efficiently in complex urban ecosystems such as parks. In Surrey, BC, Canada, Himalayan blackberry (*Rubus armeniacus*) and English ivy (*Hedera helix*) are two invasive shrub species that can negatively affect native ecosystems in cities and managed urban parks. Random forest (RF) models were created to detect these two species using a combination of hyperspectral imagery, and light detection and ranging (LiDAR) data. LiDAR-derived predictor variables included irradiance models, canopy structural characteristics, and orographic variables. RF detection accuracy ranged from 77.8 to 87.8% for Himalayan blackberry and 81.9 to 82.1% for English ivy, with open areas classified more accurately than areas under canopy cover. English ivy was predicted to occur across a greater area than Himalayan blackberry both within parks and across the entire city. Both Himalayan blackberry and English ivy were mostly located in clusters according to a Local Moran’s I analysis. The occurrence of both species decreased as the distance from roads increased. This study shows the feasibility of producing highly accurate detection maps of plant invasions in urban environments using a fusion of remotely sensed data, as well as the ability to use these products to guide management decisions.

## Introduction

Many human-dominated landscapes are invaded by non-native species, causing adverse impacts for native flora and fauna (Wilcove et al., 1998; Ehrenfeld, 2003), public health (Mack et al., 2000; Laaidi et al., 2003; Jones et al., 2009), and on ecosystem services including agricultural production, water filtration, recreation and tourism, flood mitigation, and cultural services (Pimentel et al., 2000). The decreased resilience and compromised integrity of these ecosystem services caused by invasive plant species costs at least US$34 billion annually in the United States alone (Pimentel et al., 2005; Pejchar and Mooney, 2009). Reducing the negative effects of plant invasions by restricting their spread is difficult as invasive plant species can distribute quickly through various mechanisms such as greater dispersal abilities (Kowarik, 1995; Pysek and Hulme, 2005), heightened fitness from hybridization (Ellstrand and Schierenbeck, 2000), or quicker germination (Gundale et al., 2008) than native species.

Due to the detrimental impacts of invasive plant species, city authorities are required to initiate programs to control or eradicate the plants. Notably, managers in urban areas are particularly interested in controlling invasive plants (Pysek, 1998; Pimentel et al., 2000; Pysek and Hulme, 2005), as novel habitats and increased habitat disturbance associated with urban environments provide areas where certain invasive species can thrive (Gundale et al., 2008; Lampinen et al., 2015; Hawthorne et al., 2015). In addition to being a hotspot for many plant invasions, urban ecological areas are often the most affected by them. As more people migrate to urban areas and existing urban areas are densified, ecosystem services that urban forests and other urban natural areas provide, such as increasing recreational opportunities (Arnberger, 2006), reducing the urban heat island effect and various types of pollution (Akbari et al., 2001; Escobedo et al., 2011), improving air quality (Akbari et al., 2001), reducing storm water runoff (Mcpherson et al., 2005), improving esthetics (Price, 2003), and increasing opportunities for social interaction (Kuo, 2003) will become increasingly important (Hough, 2014). It is therefore critical that the integrity of these urban natural areas is maintained, partially by controlling the spread of invasive plant species.

Detecting invasive species is typically undertaken using field surveys when field crews are available, which may be costly and have inconsistent methodologies over space and time. Additionally, field crews cannot survey large areas in sufficient detail to develop accurate maps of invasion locations. As such, different approaches are needed to monitor and map the distributions of invasive species. Remote sensing technology, specifically, light detection and ranging (LiDAR) data and hyperspectral imagery, can produce maps of the distribution of invasive plant species, augmenting previous detection approaches (Huang and Asner, 2009; He et al., 2011; Rocchini et al., 2015). LiDAR sensors are active remote sensing systems, meaning that they require and use their own energy rather than passively detecting solar energy. These sensors send pulses of electromagnetic radiation toward objects and record the time it takes for the pulse to reflect off objects and back to the sensor. Because the pulse travels at the speed of light, the distance to the object can be calculated from the return time (Bachman, 1979). In contrast, hyperspectral imagery is a passive remote sensing technology that uses the objects’ reflections of solar energy as its inputs. This type of imagery records reflectance values of an object over numerous narrow spectral channels, providing a detailed spectral signature of an object (Shaw and Burke, 2003).

Light detection and ranging data alone has been used for species habitat modeling and detection. Singh et al. (2015) mapped the distribution of an understory invasive species in urban forests using various LiDAR-derived orographic predictors as well as LiDAR-derived forest structural characteristics, such as return height variance, standard deviation, and mean to gather information about the various height strata in a stand. Some of these vegetation characteristics were found to be predictors of invasive species presence. In addition to its applications toward mapping plant distributions, LiDAR data has also been shown to be able to identify tree species and quantify the density of shrub cover using the intensity values of the LiDAR returns (Kim et al., 2009; Ørka et al., 2009; Wing et al., 2012). However, these analyses require extensive calibration (Kashani et al., 2015) and have not identified individual shrub species, thus additional information, such as detailed spectral information, may be warranted when available and when mapping distributions of shrub species.

Hyperspectral imagery excels where LiDAR data is often hindered, by offering a number of spectral channels in the visible (400–700 nm), near-infrared (700–1350 nm), and shortwave infrared (1350–2500 nm) wavelength, and narrow spectral channel widths (Adam et al., 2010). Despite the wealth of information contained in hyperspectral datasets, the applicability of using such imagery is highly dependent on the spatial resolution of the imagery and the target of analysis, as at coarse resolutions spectral signals mix and may impede fine-scale species detection (Roth et al., 2015). Peña et al. (2013) found that increasing the pixel size of hyperspectral imagery from 1.2 to 2.4 m markedly decreased the spectral separability between tree species and decreased a species classification’s accuracy by up to 25%. Early studies investigating hyperspectral detection of invasive plant species used instruments covering the visible through shortwave infrared wavelengths, yet were restricted to mapping with large (up to 20 m^2^) pixels or to mapping communities of invasive shrubs rather than specific plants (Parker Williams and Hunt, 2002; Underwood et al., 2003). More recently Underwood et al. (2006) built upon this previous research and used hyperspectral imagery with an increased spatial resolution to detect individual plants in a scrubland delta in California, USA. Hyperspectral imagery has also been applied for invasive species detection in forested areas. Asner and Vitousek (2005) mapped tree invasions by analyzing hyperspectral imagery for nitrogen concentration and relating this concentration to invasive species presence across their study area in tropical forests of Hawaii. Recent studies mapping invasive plants with hyperspectral imagery include Narumalani et al. (2009) which used airborne hyperspectral imagery focused on the visible and near-infrared wavelengths to map various species along riparian areas, Bentivegna et al. (2012) which also used airborne hyperspectral imagery, but to map an invasive shrub along highways, Ishii and Washitani (2013) and Mirik et al. (2013) which both used airborne hyperspectral imagery to map invasive herbs in grasslands, Calviño-Cancela et al. (2014) which mapped invasive plants of various lifeforms in a coastal park, and Große-Stoltenberg et al. (2016) used imagery in the visible infrared to shortwave infrared wavelengths to map an invasive tree species in dune ecosystems. While hyperspectral imagery alone has shown success detecting invasive plants, knowledge of habitat characteristics may be important in some cases for adequate mapping (Andrew and Ustin, 2008).

Fusion of LiDAR and hyperspectral technologies has also been successful in mapping invasive species, as when combined they can be used to mask certain areas or provide additional contextual environmental information. Asner et al. (2008) used hyperspectral imagery to identify and map tree species in species-rich Hawaiian rainforests and masked shaded pixels using LiDAR data. Chance et al. (2016) undertook a similar approach by masking densely forested and shaded areas using LiDAR data, to then map the distributions of two understory invasive shrub species on high spatial resolution hyperspectral images. Andrew and Ustin (2009) used hyperspectral imagery to detect an invasive plant and LiDAR data to create spatially contiguous potential habitat models from the sources of invasions, as detected by the hyperspectral imagery. This study combined LiDAR-derived metrics such as surface elevation, slope, aspect, and curvature with training field plots to produce the models. Gu et al. (2015) incorporated environmental information, and mapped gradients of species composition, including invasive species, in urban-rural gradients using hyperspectral imagery and LiDAR-derived structural variables, however, locations of individual species were not mapped.

Despite these advances in modeling and mapping invasive plant species distributions, the use of LiDAR data has been restricted to masks or structural and orographic variables when combined with hyperspectral imagery. Techniques developed for modeling direct and diffuse light regimes from LiDAR data in urban areas (see Tooke et al., 2012) may increase model accuracy by providing high resolution, relevant environmental context, in particular for plants that are sensitive to irradiance conditions (Metcalfe, 2005). Specifically, as light is a highly heterogeneous environmental condition that effects plant growth and interactions (Valladares, 2003), and as many invasive plants show preferences for certain light conditions (e.g., Metcalfe, 2005; Jansen et al., 2011), it may hold high predictive power in modeling invasive species distributions.

Another consideration when developing models with remotely sensed to detect plant distributions is deciding which type of model to utilize. While many ecological models can be developed using parametric or non-parametric regressions, they may not perform well when there are many relatively weak explanatory variables with complex interactions, as is often the case with remotely sensed data (Cutler et al., 2007). Random forests (RF) models, which are a collection of classification trees developed by selecting random subsets of the data in each, may solve this issue, as they are asserted to still be powerful when using many variables of various types (Breiman, 2001) and have been shown to outperform the majority of other classifiers in many modeling operations (Fernández-Delgado et al., 2014). Additionally, they have been used to map various invasive species with higher classification accuracies achieved compared to other models (e.g., Lawrence et al., 2006; Singh et al., 2015).

**FIGURE 1:**
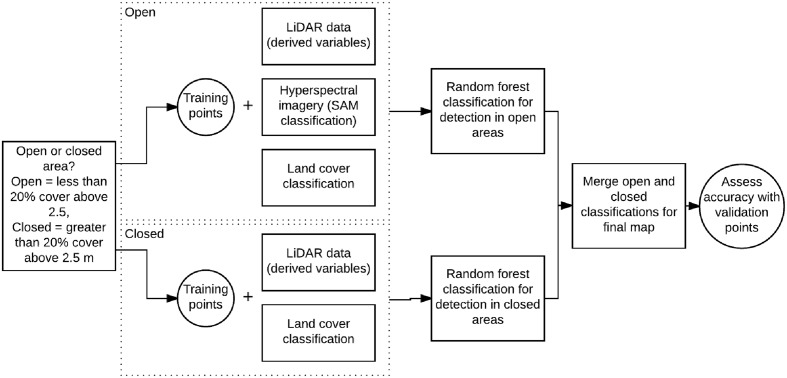
**Flow diagram showing the use of the LiDAR data, hyperspectral imagery, and field data to detect Himalayan blackberry (*Rubus armeniacus*) and English ivy (*Hedera helix*)**. SAM, spectral angle mapper.

The aim of this study is to analyze the use of a combination of LiDAR data and hyperspectral imagery to map the distributions of two invasive plant species, Himalayan blackberry (*Rubus armeniacus*) and English ivy (*Hedera helix*), in the urban area of Surrey, British Columbia, Canada. First, a spectral detection algorithm using hyperspectral imagery was performed across open areas of the city to map the spectral closeness of each pixel compared to the two species (**Figure 1**). Second, LiDAR data was utilized to create a map of local solar irradiance conditions during the growing season in vegetated areas across the city. Lastly, a map from a RF model was produced with LiDAR-derived environmental variables, the results of the spectral classification, and the irradiance models as inputs to map Himalayan blackberry and English ivy across the entire city at a 1.0 m pixel resolution. The accuracies of the maps were assessed, the importance of model variables and spatial information were analyzed, and implications were discussed.

## Study Area and Species

**FIGURE 2:**
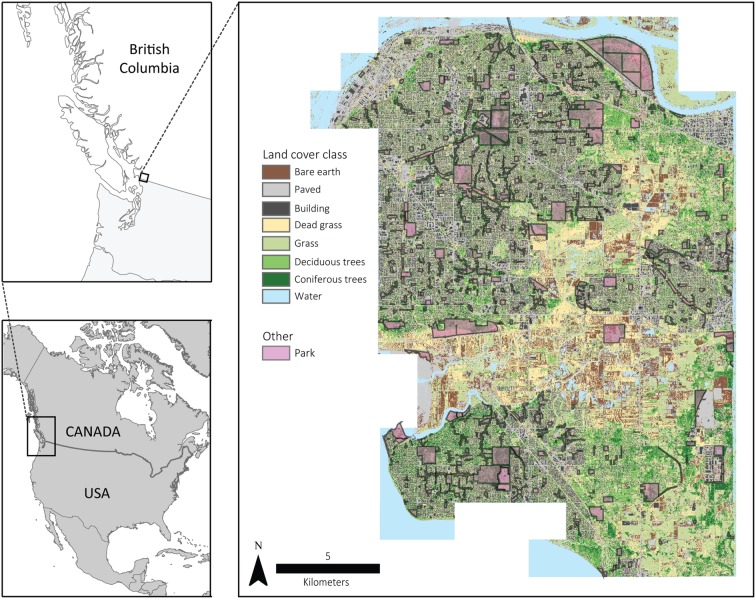
**Entirety and location of the Surrey, BC, Canada, the land cover classification from Plowright et al. (in review) and the municipal parks system across the city**.

Surrey (49°11′N, 122°51′W) is located in the greater Vancouver area of British Columbia, Canada (**Figure 2**), covers 316 km^2^, and contains a mosaic of land cover patches including urban, agricultural, forested, wetland, and grassland area. Vegetated areas often contain dense, shrubby and herbaceous understories, as is commonly found in the temperate coastal climate of the area. Additionally, the city contains a mixture of public and private lands. On public lands, city management maintains an active parks and natural areas (**Figure 2**) system that contains many of the aforementioned land cover types. These land cover types are also present on private lands.

Himalayan blackberry (*Rubus armeniacus*) and English ivy (*Hedera helix*) are two invasive shrub species that land managers aim to control, due to their harmful effects on biodiversity, city residents, and users of natural areas. Himalayan blackberry displaces native vegetation and birds in both disturbed and undisturbed areas (Amor, 1973; Caplan and Yeakley, 2006; Astley, 2010), and alters successional patterns along streams (Fierke and Kauffman, 2006). Individual Himalayan blackberry plants grow and reproduce quickly, producing large amounts of seeds, and outcompete native plants (Schwartz et al., 1996; Caplan and Yeakley, 2006). Plants overgrow walking paths and roadways, creating a nuisance for city residents and managers. Himalayan blackberry generally grows near sea level in moist sites, in a variety of conditions from pastures to riparian areas and forest edges (Gaire et al., 2015). English ivy may produce allergens (Jones et al., 2009; Paulsen et al., 2010), has competitive advantages over native plants (Thomas, 1980; Dlugosch, 2005), and endangers users of natural areas by weakening trees (Thomas, 1980). English ivy also decreases light levels in the understory, resulting in reduced cover of native herbs and shrubs in the understory (Thomas, 1980; Harmer et al., 2001; Dlugosch, 2005), and may be a deterrent to wildlife (Barnea et al., 1993). It is challenging to control due to the many various life forms it can assume: herbaceous vines, climbers, herbs, woody shrubs, and sometimes small trees (Metcalfe, 2005). It is mostly a forest-dwelling species, but is common in open areas as well (Clergeau, 1992).

## Materials and Methods

### Field Measurements

The presence and absence of Himalayan blackberry, English ivy, and other common plant species were recorded by City of Surrey field crews throughout 2012 and 2013 in park natural areas. The municipal government sent experts to locate invasive species across the city according to a stratified design that targeted areas known to be susceptible to invasions first. These initial surveyed areas included forest edges, riparian areas, fields, edges to developed properties, and trails. Following the survey of these selected sites, other locations in parks and natural areas across the city were surveyed for invasions. Single points in large, discrete, established patches dominated by the invasive species with an area of greater than 40 m^2^ separated by least 10 m from another plot were recorded as having either a presence or absence of the two species. These plots also contained a clear dominance of the invasive species over all species. An eTrex 10 GPS device (Garmin, Olathe, KS, USA) with a dGPS positional accuracy within 3 m was utilized. After the field survey, plots were randomly categorized as either training or validation, creating 646 training plots (108 Himalayan blackberry, 123 English ivy, and 415 of other species), and 496 validation (160 Himalayan blackberry, 114 English ivy, and 222 of other species) which were used for detection models.

### Remotely Sensed Data

Between April 3 and April 11, 2013, airborne LiDAR data were collected for the entire City of Surrey (**Figure 2**) by Airborne Imaging (Calgary, AB, Canada). The Leica ALS70-HP (Leica Geosystems, Heerbrugg, Switzerland) system sent pulses with a wavelength of 1064 nm from an altitude of 1000 m with a view angle of 20° and a scan frequency of 53 kHz resulting in a point density of 25 points/m^2^. TerraScan software (Terrasolid, Helsinki, Finland) classified points as ground or non-ground. TerraScan processing was completed using standard default parameters (Kraus and Pfeifer, 1998; Axelsson, 1999).

The Compact Airborne Spectrographic Imagery (CASI) 1500 acquired hyperspectral imagery over Surrey on May 4, 2013 at a 1.0 m pixel resolution. The imagery consisted of 72 spectral channels from 363 to 1051 nm with a constant channel width of 9.6 nm. Following the acquisition of the images, they were radiometrically corrected, georeferenced, and converted to reflectance imagery. To radiometrically correct the imagery, raw digital numbers, value representations of the contents of a pixel, were converted into spectral radiances for each spectral channel based on calibrations from ITRES Research (Calgary, AB, Canada), who calibrated the radiance imagery to known lab calibration files and corrected for dark and electronic offsets. These corrected images were georeferenced to a 1.0 m digital elevation model (DEM) produced from the LiDAR data and ground-based global positioning data (GPS) data. Lastly, atmospheric conditions and topographic and bi-directional effects were corrected to produce reflectance imagery that ranges from 0 to 100% using the ATCOR-4 procedure (Richter and Schlapfer, 2016).

**FIGURE 3:**
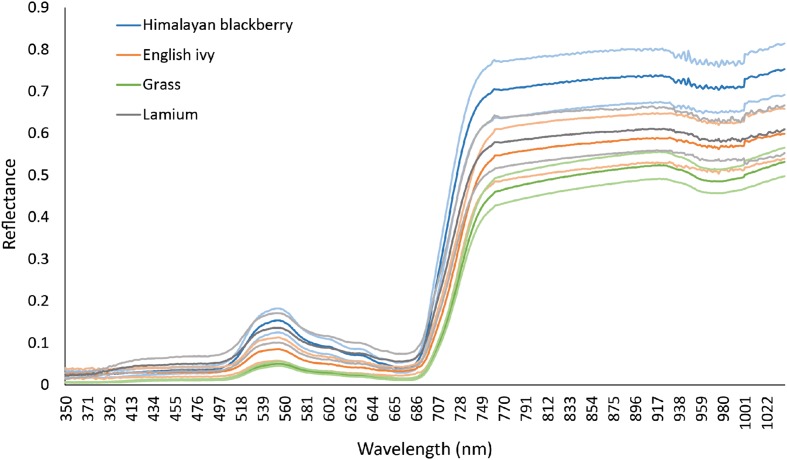
**Reflectance of Himalayan blackberry (*Rubus armeniacus*), English ivy (*Hedera helix*), grasses, and lamium (*Lamium galedo*…) in Surrey, BC, Canada as obtained from an Analytical Spectral Devices spectrometer in the spectral range of the hyperspectral imagery**. Mean reflectance is indicated by dark lines, and ±1 standard deviation is indicated by the lighter lines.

Spectra of the two species of interest, Himalayan blackberry and English ivy, and other common shrub or herbaceous species in open areas in Surrey, lamium (*Lamium galeobdolon*) and grasses (lawn and European beachgrass [*Ammophila arenaria*]), were collected in April 2015 using a portable Analytical Spectral Devices (ASD) full range (FR) spectrometer (Analytical Spectral Devices, Boulder, CO, USA) within ±2 h from solar noon while the leaves were still attached. This spectrometer recorded spectra from 350 nm to 2500 nm at a 3 nm channel resolution up to 1000 nm and a 10 nm channel resolution at wavelengths between 1000 and 2500 nm. To account for differing light conditions between samples, the spectrometer was calibrated before each measurement with a Spectralon panel (Labsphere, North Sutton, NH, USA). At each measurement, the sensor was held approximately 10 cm from the surface of the plant, and leaves were manipulated to be stacked at least six deep, to achieve a standard optical depth (Datt, 1998; Jones et al., 2010). While 10 cm away from the plant, the sensor was moved around in circles to acquire spectra from various parts of the plant simultaneously, providing spectra that were representative of the entire plant from the view on the imagery. This sampling protocol controlled for variable irradiance conditions between samples by calibrating for the irradiance at each sample with the Spectralon panel and by limiting our sample area to the small area covered by the 10 cm circles. Between 10 and 15 samples were collected for each species. After measuring spectra, ASD channels were converted to the 72 CASI channels via averaging (Chance et al., 2016). **Figure 3** shows the spectra of these species prior to resampling.

### Creation of Model Variables

#### LiDAR-Derived Variables

As orography and forest structural characteristics may partially drive understory plant invasions (Royo and Carson, 2006), and as some invasive plant species, particularly shrub species, can alter forest structural characteristics (e.g., Hartman and McCarthy, 2008; Kuebbing et al., 2014), numerous orographic and forest attribute layers were created. Summaries of the vertical LiDAR data distribution were produced across the entire extent of the city, at a 1.0 m pixel resolution. The following layers were created using FUSION software (version 3.42, United States Forest Service, Seattle, WA, USA): a digital elevation model (DEM) of the elevation of the ground, a digital surface model (DSM) of the elevation of the first returns including trees and buildings, 75th (P75), 90th (P90), and 95th (P95) height percentiles, kurtosis of height, skewness of height, coefficient of variation of height, canopy cover and penetration above a height of 2.5 m, and cover below a height of 2.5 m (**Table 1**). As height percentiles are a function of the locations of points vertical profiles, increased understory cover due to plant invasions may decrease the height percentile values when comparing forests with similar structure and height otherwise. Metrics that describe vertical point distributions were included, as kurtosis of height was found to be negatively related to an invasive understory plant in Singh et al. (2015). For cover and penetration, a height of 2.5 m was chosen as a threshold as Himalayan blackberry and English ivy plants that are not vines on trees mostly occur below this height in Surrey.

**Table 1:** Variables used as inputs to the random forest models to detect Himalayan blackberry (*Rubus armeniacus*) and English ivy (*Hedera helix*) in Surrey, BC, Canada.

Variable type	Variable name	Description
Orographic	Digital elevation model (DEM)	Ground height from LiDAR returns
	Digital surface model (DEM)	Surface heights from LiDAR returns
	Aspect	“Northness,” 1 being north and -1 being south of a pixel on the DEM
	Slope	Slope of a pixel on the DEM
	Curvature	Degree of concavity of a pixel on the DEM
	Plan curvature	Degree of concavity perpendicular to the maximum slope
	Profile curvature	Degree of concavity parallel to the maximum slope
	Topographic wetness index	Wetness based on hydrological accumulation as modeled by the slope and contributing area, and as described by Beven and Kirkby (1979).
Vegetation attributes	95th percentile height (P95)	Height of the 95th percentile of LiDAR returns
	90th percentile height (P90)	Height of the 90th percentile of LiDAR returns
	75th percentile height (P75)	Height of the 75th percentile of LiDAR returns
	Kurtosis	Kurtosis of the height of LiDAR returns
	Skewness	Skewness of the height of LiDAR returns
	Coefficient of variation	Coefficient of variation of height of LiDAR returns
	Penetration above a height of 2.5 m	Proportion of total LiDAR returns in a pixel above a height of 2.5 m
	Cover below a height of 2.5 m	Proportion of total LiDAR returns in a pixel below a height of 2.5 m that are also above ground
	Distance to open area	Distance to area with less than 10% canopy cover
Spectral (only applies to open areas)	Himalayan blackberry rule image (all channels)	SAM rule image of Himalayan blackberry using all spectral channels
	Himalayan blackberry rule image (channel subset)	SAM rule image of Himalayan blackberry using a subset of spectral channels
	English ivy rule image (all channels)	SAM rule image of English ivy using all spectral channels
	English ivy rule image (channel subset)	SAM rule image of English ivy using a subset of spectral channels
Land cover classification and products	Land cover classification (from Plowright et al. (in review))	Seven class land cover classification from the LiDAR data and hyperspectral imagery
	Distance to grass	Distance to grass as determined by the land cover classification
	Distance to impervious	Distance to impervious surfaces as determined by the land cover classification
Irradiance layers	Direct irradiance	Average daily direct irradiance from the 15th day of each month of the growing season at 3 m by 3 m resolution across Surrey
	Diffuse irradiance	Average daily diffuse irradiance from the 15th day of each month of the growing season at 3 m by 3 m resolution across Surrey

*All variables are rasters with a pixel size of 1.0 m except for the irradiance layers, which have pixel sizes of 3.0 m*.

Orographic variable layers were created in ArcMap (version 10.3, Esri, Redlands, CA, USA). Slope and aspect were calculated from the DEM. A cosine transformation was applied to aspect to convert the value range from -1 to 1, 1 being north (360°), and -1 being south (180°). Curvature rasters, which describe the concaveness of a surface, were created from the DEM. From the penetration layer, a measure of distance to open areas was created for each pixel. Pixels with penetration values above 90% were classified as open areas. Distances to these areas were calculated using Euclidean Distance. Lastly, a topographic wetness index, which is a unitless index quantifying hydrological accumulation based on slope and contributing area (Sørensen et al., 2006), was produced (Beven and Kirkby, 1979; **Table 1**).

#### Invasive Species and Land Cover Classifications

Rule images from a spectral angle mapper algorithm (SAM; Kruse et al., 1993) for Himalayan blackberry and English ivy were produced using the hyperspectral imagery in open areas (all areas with less than 20% canopy cover above a height of 2.5 m). SAM algorithms are well-established spectral matching algorithms that classify pixels based on the angle between spectral vectors. Rule images indicate the degree to which the spectral signature of a pixel matches the spectral signature of the target object. Smaller differences between angles represent more closely aligned spectra. Spectra from 73 of the field plots in open areas (38 of Himalayan blackberry and 35 of English ivy) were extracted from the hyperspectral imagery. As hyperspectral imagery can be processing intensive and contain redundancy in the spectral signals across channels (Clark et al., 2005), a spectral channel selection was applied to test whether or not all channels were needed for accurate classification. Chance et al. (2016) identified key spectral channels for differentiating Himalayan blackberry and English ivy from lamium and grasses, other common species in Surrey, and each other, using the instability index (ISI; Somers et al., 2010) channel selection and spectra from the ground-based acquisition. **Table 2** describes these spectral channels. Prior to producing SAM rule images, forested areas were omitted by eliminating pixels with more than 20% cover above a height of 2.5 m according to the LiDAR-derived canopy cover layer. SAM rule images were produced by classifying the hyperspectral imagery using both the channel subsets and all channels of the spectra for Himalayan blackberry and English ivy across the open areas of the city in ENVI software (version 5.1, Exelis, McLean, VA, USA).

**Table 2:** Key CASI (Compact Airborne Spectrographic Imagery) spectral channels and their corresponding wavelengths for differentiating Himalayan blackberry (*Rubus armeniacus*) and English ivy (*Hedera helix*) from each other and other common species in Surrey, BC, Canada, and the possible causes of the responses shown at these wavelengths and discussed in Chance et al. (2016).

Species	Wavelength at channel center (nm)	Corresponding CASI channel number	Possible reason for response as discussed in Chance et al. (2016)
Himalayan blackberry	512	16	Nitrogen, phosphorus, potassium or a combination of these (Mutanga et al., 2004)
	559	21	Lack of nitrogen or potassium (Mutanga et al., 2004)
	655	31	Electron transition response to Chlorophyll a (Curran, 1989) and/or red edge beginning
	712	37	
	750	41	End of red edge
	922	59	
	960	63	
	979	65	
	1008	68	
	1027	70	Water, cellulose, starch or lignin (Curran, 1989)
English ivy	569	22	Lack of nitrogen or potassium (Mutanga et al., 2004)
	588	24	
	607	26	
	693	35	Beginning of red edge
	741	40	End of red edge
	855	52	
	912	58	
	970	64	

As land cover type may be an indicator for plant invasions (see Roy et al., 1999; Pearson et al., 2004), a land cover map of Surrey established by Plowright et al. (in review) was used as a categorical input in the detection models (**Figure 1**). Seven classes, coniferous forest, deciduous forest, grass, bare earth, paved areas, buildings, and water were classified at a 1.0 m pixel resolution using LiDAR data and hyperspectral imagery. The overall accuracy of this land cover classification was 88.6% (Plowright et al., in review). The resulting total surface area of pervious surfaces (coniferous forest, deciduous forest, grass, and bare earth) was 215 km^2^, 68% of the total area of Surrey.

#### Irradiance Model

Because invasive plant distributions may be related to light levels, especially in forests in western North America (Parendes and Jones, 2000), an irradiance model indicating the light regime across the city was developed. Methods established by Tooke et al. (2012) were used to map irradiance at 3 m by 3 m pixel resolution. In this procedure, the LiDAR returns, the DSM, and the DEM were used in conjunction with solar angles and typical atmospheric conditions to calculate direct and diffuse irradiance on the 15th day of each month between March and October in 2013. Direct irradiance at each hour from 5 am to 10 pm local time was calculated according to the following three steps: atmospheric transmission, viewshed calculation, and vegetation transmission. Prior to taking these steps, the solar position was calculated according to the ENEA solar position algorithm (Grena, 2008) for each time interval.

The atmospheric transmission step calculates the irradiance that penetrates the atmosphere, a function of turbidity and cloud cover. Monthly broad scale (50 km) global turbidity maps were obtained from the National Oceanic and Atmospheric Administration website and the values over Surrey were used as inputs into the model. A clearness index Hammer et al. (2003), describing light transmission through clouds of 0.5, was applied to the model based on values obtained in Tooke et al. (2012) from a nearby weather station.

Second, a viewshed of the effective horizon from the point 2.5 m above the DEM was produced for 36 viewing azimuths. At each pixel and azimuth, the angle of the tallest obstruction within 100 m, whether tree, building, or otherwise, was obtained from the DSM, relative to the orientation and slope of the pixel. This information was used to calculate the sky view factor (SVF), a measure of the directly visible proportion of the sky.

Within vegetated pixels, an extinction coefficient, calculated from the proportion of LiDAR returns at regular height intervals, was determined. A two-parameter Weibull distribution function, previously shown to be capable of characterizing vegetation structure from LiDAR returns (Coops et al., 2007; Tooke et al., 2012), was produced at each using a vertical scaling parameter, a shape parameter, and the extinction coefficient as inputs. The vertical scaling parameter and shape parameter were estimated using a Levenberg-Marquardt least-squares analysis for LiDAR returns in each pixel (Tooke et al., 2012). The extinction coefficient was the proportion of LiDAR returns above a certain height (Tooke et al., 2012). These parameters varied across the study area as they were determined by the vertical distribution of LiDAR returns at each pixel.

The diffuse irradiance component was calculated using the diffuse transmissivity function and solar altitude function from Hofierka et al. (2002), which depends on atmospheric turbidity and solar elevation, in conjunction with the SVF (see Tooke et al., 2012, Eq. 9). The two output layers were the average daily diffuse and direct irradiance during the growing season from March to October with units of MJ m^-2^ day^-1^.

### Detection Model

#### Development

Four binary RF detection models were developed for each species in R programming environment (version 3.1.2, R Core Team) using the randomForest package (Liaw and Wiener, 2002) with 2000 trees and default values for the number of variables selected at each node and all other input parameters. Two models detected Himalayan blackberry and English ivy respectively in open areas using SAM rule images and LiDAR-derived variables relevant to open areas, and the other models detected the species in areas of canopy cover using only LiDAR-derived variables. In this present study, the model development processes were iterative, as initially all relevant remote sensing predictor variables (**Table 1**) were considered. Using the training data, correlated and unimportant variables were iteratively removed until only uncorrelated variables with high predictive power remained, using mean decrease accuracy provided by the RF models as the measure of predictive power. Of variables that were correlated, the variable with higher predictive power was included in the model. Variables with negative mean decrease accuracies were excluded. Prior to fitting the model, two masks were created. Pixels with less than 10% cover below a height of 2.5 m were omitted and dirt, paved areas/rock, buildings, and water were masked using the land cover classification. The RF models were applied to the unmasked areas only, and produced maps of presence and absence of Himalayan blackberry and English ivy across the study area. For both species, the resulting maps of open areas and areas of closed canopy were combined to produce one map for each species or its distribution across the entire city.

According to Breiman (2001), RF classifications do not require independent accuracy assessments as some training data is inherently used as validation data to produce unbiased error. However, using this implicit error estimate may inflate the accuracy of the classification in some cases (Millard and Richardson, 2015). For detecting Himalayan blackberry and English ivy, the validation data from field plots were used to conduct an independent accuracy assessment. A minimum mapping unit (MMU) of 3 m radius circles around the validation plots was established since the two irradiance layers had pixels of 3 m resolution. Detection of the species of interest, either Himalayan blackberry or English ivy, within one of these plots counted as a presence in the accuracy assessment, indicating a true positive. Detection in an absence plot was recorded as a presence, and thus a false positive. The same logic applied for true and false negatives. Accuracies, kappa coefficients, and true skill statistics (Allouche et al., 2006) were assessed accordingly from the output map.

The uncertainty associated with accuracy was assessed as well at each validation point. RF models can provide spatial information about the probability of a pixel being classified as a certain class (either presence or absence in this present study; Cutler et al., 2007). This RF probability metric can be utilized to derive information about the classification uncertainty (Loosvelt et al., 2012). In this present study, *U* = 1 – *p*_max_ was calculated for each pixel, where *p*_max_ was the probability that a pixel was most likely to occur in its class based on the RF model. A higher value indicated class confusion and a lower value indicated greater certainty about the result (Loosvelt et al., 2012). In this present study, the maximum probability within the MMU that corresponded to the same class as the validation plot was used to assess the uncertainty.

#### Analysis

Variable importance was provided by the RF algorithm. The map was used to quantify the distributions of Himalayan blackberry and English ivy across the extent of the city and within parks. The total areas of Himalayan blackberry and English ivy were determined across the entire city and in parks according to the number of 1 m by 1 m pixels classified as the respective species. For analyzing the distribution of the species across the parks system, the number of parks containing the species was tabulated. Additionally, ecological relationships were analyzed by extracting information from the LiDAR-derived layers at all the field plots, training and validation. For this analysis, field plots were one of six categories: Himalayan blackberry open, Himalayan blackberry closed, English ivy open, English ivy closed, absence open, or absence closed. Values of the variables were compared between open and closed areas, as well as species presence and absence.

As the distribution of invasions across the city has management implications, clusters and outliers of Himalayan blackberry and English ivy were identified using Anselin Local Moran’s I. Moran’s I is an indicator used to quantify spatial autocorrelation (Moran, 1950; Anselin, 1995). Anselin Local Moran’s I, which measures local autocorrelation, has proven to be an effective indicator of spatial clustering in ecological studies (see Fu et al., 2014; Swetnam et al., 2015). This metric ranks the abundance of species in each cell of a grid and determines which areas are significantly clustered or separated. A grid of cells 100 m by 100 m was applied across the entire study. At each cell in this grid, the number of pixels with Himalayan blackberry or English ivy invasion was tabulated, creating a measure of abundance. Anselin Local Moran’s I was calculated across the city with α = 0.05 for both Himalayan blackberry and English ivy.

## Results

### Models

**FIGURE 4:**
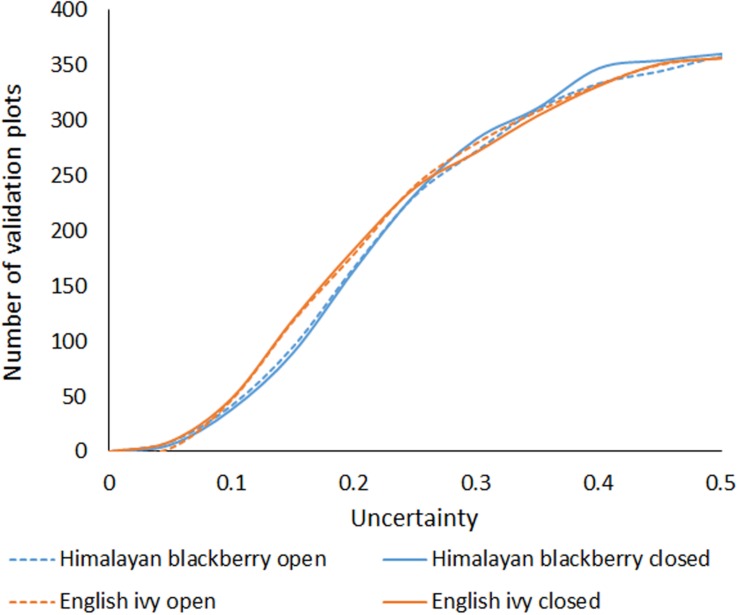
**Cumulative sum of uncertainty, calculated by subtracting the random forests probability from 1, for each classification at all of the validation points**. Lower values are more certain.

The overall accuracies of the invasive species detection models ranged from 77.8 to 87.8% (**Table 3**). Himalayan blackberry in open areas was detected best, followed by English ivy in open areas (areas with canopy cover less than 20% above a height of 2.5 m), English ivy in areas with closed canopies (areas with greater than 20% canopy cover above a height of 2.5 m), and Himalayan blackberry in areas with closed canopies (**Table 3**). Detection of Himalayan blackberry in open areas had the highest kappa coefficient (0.75) followed by English ivy in open areas (0.56), Himalayan blackberry in areas with closed canopy (0.55), and English ivy in areas with closed canopy (0.53; **Table 3**). True skill statistics took a different order, with Himalayan blackberry in open areas receiving the highest (0.78), followed by Himalayan blackberry in areas with closed canopy (0.55), the English ivy in open areas (0.52) and areas with closed canopy (0.48; **Table 3**). Himalayan blackberry was detected with higher true positive rates (sometimes referred to as sensitivity) than English ivy in both open areas and areas with closed canopies (**Table 3**). English ivy was detected with higher true negative rates (sometimes referred to as specificity) than Himalayan blackberry in both open areas and areas with closed canopies (**Table 3**). False negative rates (miss rates) were higher for English ivy than for Himalayan blackberry and false positive rates (fall-out) were higher for Himalayan blackberry than English ivy (**Table 3**). All classifications had low uncertainty, as the majority of validation points showed uncertainty values below 0.25 (**Figure 4**). Himalayan blackberry in areas with closed canopies had the highest uncertainty (**Figure 4**).

**Table 3:** Accuracy of the random forest models for detecting Himalayan blackberry (*Rubus armeniacus*) and English ivy (*Hedera helix*) in open areas and areas with closed canopies in Surrey, BC, Canada.

		Open				Closed			
		Himalayan blackberry		English ivy		Himalayan blackberry		English ivy	
		Observed				Observed			
		Presence	Absence	Presence	Absence	Presence	Absence	Presence	Absence
Predicted	Presence	42	12	23	6	81	21	41	11
	Absence	3	66	16	78	34	112	34	162
	Overall accuracy (%)	87.8		82.1		77.8		81.9	
	True positive rate (sensitivity; %)	93.3		59.0		70.4		54.7	
	True negative rate (specificity; %)	84.6		92.9		84.2		93.6	
	False negative rate (miss rate; %)	6.7		41.0		29.6		45.3	
	False positive rate (fall-out; %)	15.4		7.1		15.8		6.4	
	Kappa	0.75		0.56		0.55		0.53	
	True skill statistic	0.78		0.52		0.55		0.48	

*Presence and absence data are in number of points collected*.

**FIGURE 5:**
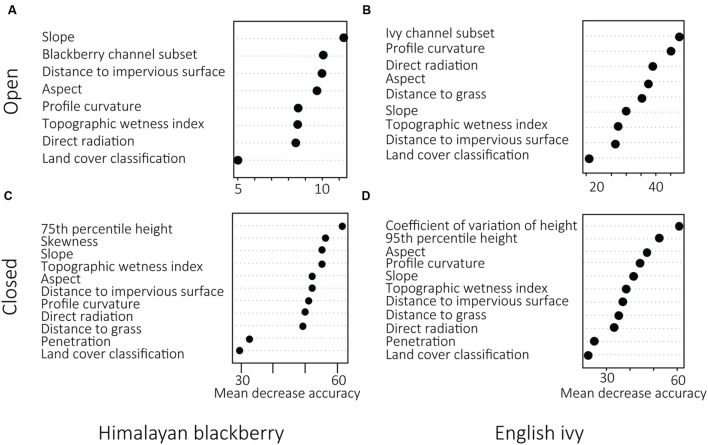
**Variable importance as expressed by mean decrease accuracy (% decrease in overall accuracy) determined by random forest classifications in open areas **(A,B)** and areas with closed canopies **(C,D)** for Himalayan blackberry (*Rubus armeniacus*) and English ivy (*Hedera helix*) distributions across Surrey, BC, Canada**.

**FIGURE 6:**
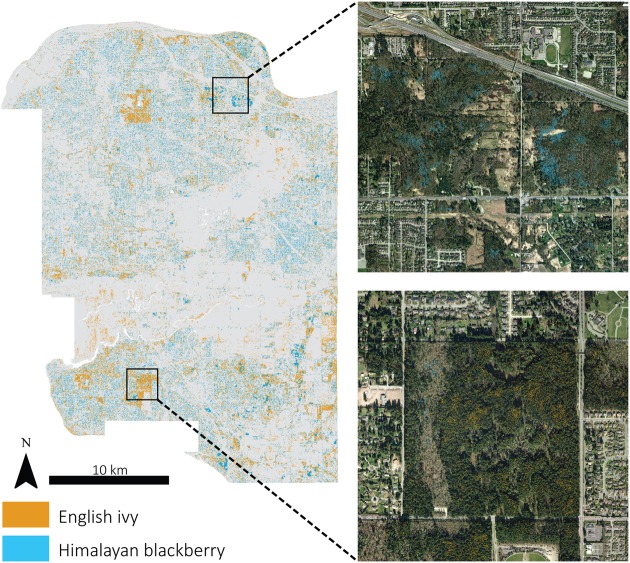
**Detected locations of Himalayan blackberry (*Rubus armeniacus*) and English ivy (*Hedera helix*) across Surrey, BC, Canada displayed with a 7 m filter and subsets with actual detected areas**.

In open areas, the SAM rule images were ranked as highly important for both Himalayan blackberry and English ivy (**Figure 5**). For both species, the SAM rule image produced using the subset of spectral channels was chosen over the SAM rule image from all spectral channels (**Figure 5**). Direct irradiance was more important to English ivy detection than to Himalayan blackberry detection in open areas (**Figure 5**). In areas with closed canopies, the most important predictor variables – 75th and 95th percentile height, skewness, and the coefficient of variation of height – were related to forest structural characteristics (**Figure 5**). Aspect was an important variable in both open areas and areas with closed canopies for both species (**Figure 5**). Land cover classification was ranked as the least important variable in all classifications, however, its product, distance to impervious surface, and was ranked highly for detecting Himalayan blackberry in open areas (**Figure 5**). **Figure 6** shows examples of the final classification results.

### Distribution of Invasive Species

Across the city, Himalayan blackberry covered 1.18 km^2^ and English ivy covered 1.51 km^2^, corresponding to 0.5 and 0.7% of the pervious areas respectively (**Table 4**). Anselin Local Moran’s I results showed that Himalayan blackberry and English ivy were similarly clustered, with 98.5 and 99.3% of 100 m by 100 m grid cells containing either of the species located in clusters (**Table 5**). 1.5% of grid cells with Himalayan blackberry and 0.7% of grid cells with English ivy were spatial outliers (**Table 5**). Within parks, Himalayan blackberry covered 0.16 km^2^ and English ivy covered 0.35 km^2^, corresponding to 0.8 and 1.8% of the pervious surface respectively (**Table 4**). Although the area covered by English ivy was greater than Himalayan blackberry in parks, Himalayan blackberry was present in more parks (**Table 4**).

**Table 4:** Total area covered across the parks system and the city, and number of parks and 10 m by 10 m cells covered by English ivy (*Hedera helix*) and Himalayan blackberry (*Rubus armeniacus*) in Surrey, BC, Canada.

		Himalayan blackberry	English ivy
Parks	Total area (km^2^)	0.16	0.35
	Proportion of parks with occurrence	90.8	88.3
City	Total area (km^2^)	1.18	1.51

*Number of cells (*N*) = 1179195 for Himalayan blackberry and *N* = 1507252 for English ivy*.

**Table 5:** Percent of area invaded by Himalayan blackberry (*Rubus armeniacus*) and English ivy (*Hedera helix*) in significant clusters and outliers as determined by Anselin’s Local Moran’s I in Surrey, BC, Canada.

Percent of invaded area	Himalayan blackberry	English ivy
In clusters	98.5	99.3
In outliers	1.5	0.7

**FIGURE 7:**
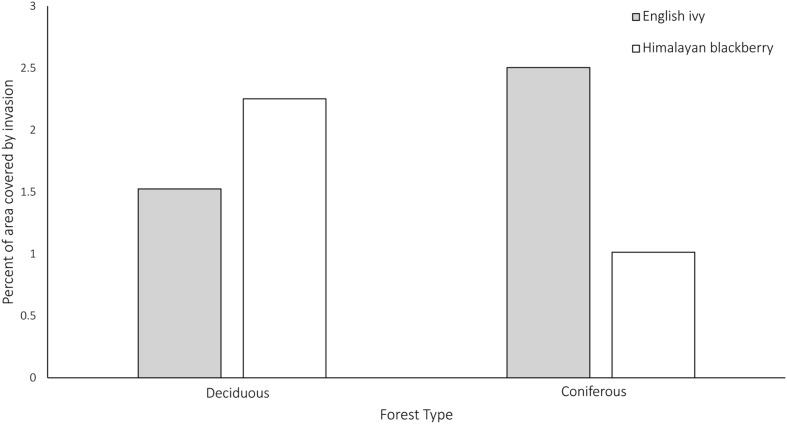
**Percent of area covered by Himalayan blackberry (*Rubus armeniacus*) and English ivy (*Hedera hel*ix) in deciduous and coniferous forests in Surrey, BC, Canada**. *N* = 1179195 for Himalayan blackberry and *N* = 1507252 for English ivy.

**FIGURE 8:**
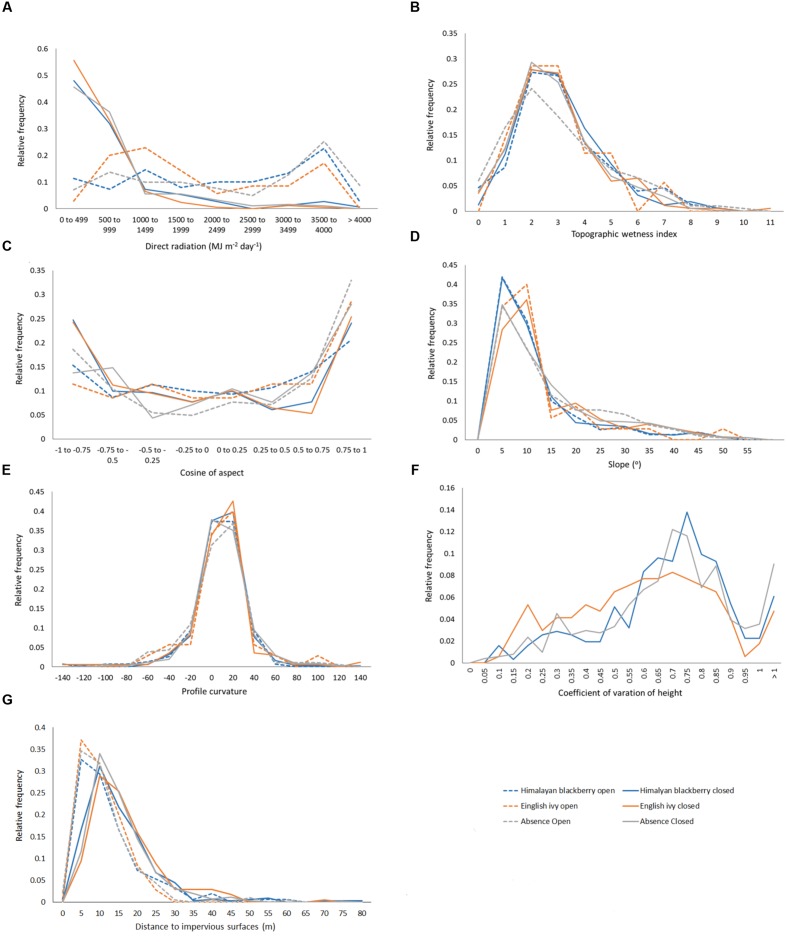
**Relative frequencies of values in Himalayan blackberry (*Rubus armeniacus*) and English ivy (*Hedera helix*) presence and absence plots for **(A)** direct radiation, **(B)** topographic wetness index, **(C)** cosine of aspect (“northness”), **(D)** slope, **(E)** profile curvature, **(F)** the coefficient of variation of height, and **(G)** the distances to impervious surfaces in Surrey, BC, Canada**.

In addition to differing in their spread across the city and parks systems, Himalayan blackberry and English ivy invasions differed in their relationships to forest types, irradiance, and roads. Himalayan blackberry was found more often in deciduous areas with closed canopies than in coniferous areas with closed canopies (**Figure 7**). The opposite was true of English ivy. In both open areas and areas with closed canopies, Himalayan blackberry did not have a strong relationship to direct irradiance compared to absence plots, whereas English ivy occurred at areas with less direct irradiance (**Figure 8**). Himalayan blackberry and English ivy in areas with closed canopies followed similar trends in regards to the cosine of the aspect (**Figure 8**). Both species had higher relative frequencies at most south facing aspects than absence plots, and vice versa for north facing aspects (**Figure 8**). For both species in open areas, there were relatively fewer occurrences at extreme north and south aspects compared to absence plots, yet there were relatively more occurrences at east or west aspects when compared to absence plots (**Figure 8**). For both species in both areas, the majority of profile curvature values occurred between 0 and 20, which corresponds to slightly concave surfaces (**Figure 8**). Topographic wetness for both species in both areas were skewed toward drier values (**Figure 8**). Slope was skewed toward lower values; however, English ivy in open areas and areas with closed canopies occurred most frequently at slopes between 5 and 10 degrees, whereas Himalayan blackberry and absences occurred mostly at slopes between 0 and 5° (**Figure 8**). Himalayan blackberry had a peak coefficient of variation of height at 0.8, whereas English ivy had a peak at 0.7 (**Figure 8**). English ivy had a coefficient of variation of height more skewed toward smaller values than Himalayan blackberry (**Figure 8**). Both presences and absences for both species in open areas were closer to impervious surfaces than in areas with closed canopies (**Figure 8**).

**FIGURE 9:**
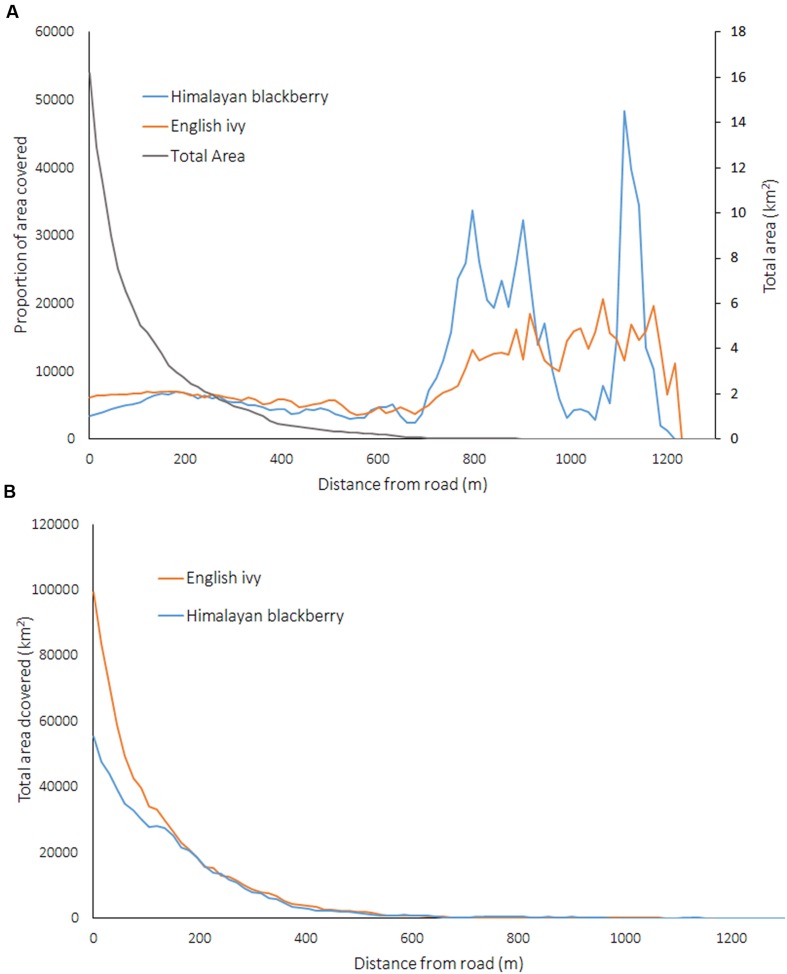
**(A)** Proportion covered by Himalayan blackberry (*Rubus armeniacus*) and English ivy (*Hedera helix*) and the total area covered in the city related to the distance from roads, and **(B)** total area covered by Himalayan blackberry (*Rubus armeniacus*) and English ivy (*Hedera helix*) related to the distance from roads in Surrey, BC, Canada.

The absolute amount of Himalayan blackberry and English ivy decreased as the distance from roads increased, yet when this area was considered proportionally to the land area at various distances from roads, there were no relationships between invasive species occurrence and the distance to a road at areas less than 500 m from a road (**Figure 9**). Areas further than 500 m away from road had proportionally higher rates of invasion than areas within 500 m of a road (**Figure 9**).

## Discussion

Few studies have mapped the distribution of invasive shrub species in complex urban areas using remotely sensed products. In one of these studies, Singh et al. (2015) mapped an invasive shrub, Chinese privet (*Ligustrum sinense*), in Charlotte, North Carolina at a 5 m by 5 m resolution using LiDAR data and a RF classifier and achieved overall accuracies between 81 and 89%. The results of this present study were similar to those found in Singh et al. (2015) with overall classification accuracies of 77.8 and 87.8% (**Table 3**). The true skill statistic, a metric of classification performance that is independent of prevalence, which the kappa coefficient and overall accuracy are not (Allouche et al., 2006), showed that Himalayan blackberry was actually better classified than English ivy in all cases due to the low true positive rates in the English ivy classifications (**Table 3**). The relatively low true positive rates for English ivy in both open areas and areas with closed canopy indicate that the RF classifier may have underestimated the area covered by English ivy. The low true positive rates may also indicate that the classifier confused English ivy with other species surveyed in Surrey, and the estimated area may not be inaccurate. However, English ivy is more specialist than Himalayan blackberry (Metcalfe, 2005; Gaire et al., 2015), thus the second possibility is less likely considering the high accuracies of the Himalayan blackberry classifications. Additionally, the uncertainty analysis showed that Himalayan blackberry classifications actually had higher uncertainties that the English ivy classifications (**Figure 4**), indicating that instead of confusing English ivy with other species the RF classifier was likely to underestimate the species range for other reasons. As English ivy covered more areas in parks and across the city than Himalayan blackberry, but was present in a proportionately smaller number of parks (**Table 4**), English ivy was likely more clustered than Himalayan blackberry. Anselin Local Moran’s I results did not corroborate this, however, as the number of 100 m by 100 m grid cells that were considered parts of Himalayan blackberry or English ivy clusters were similar for the two species (**Table 4**). It is logical that English ivy was clustered when considering its habitat preferences. As English ivy prefers coniferous forests (Clergeau, 1992; **Figure 7**), its potential range was limited to a few patches across the city.

Open areas were classified with higher accuracies than areas with closed canopies for both Himalayan blackberry and English ivy. This is likely due to the inclusion of the rule images from the hyperspectral imagery in the model. The SAM rule images provided a continuous variable on which the RF model could make decisions. Other SAM classifications, such as that classifying Himalayan blackberry and English ivy in Chance et al. (2016), transform the continuous rule image into a binary classification image by defining a threshold SAM value, below which is classified as presence and above which is classified as absence. By keeping the classification results as continuous, the RF model had greater flexibility in defining this threshold based on the values of the other predictor variables. For both Himalayan blackberry and English ivy, the SAM rule images were highly ranked as important (**Figure 5**). More specifically, the SAM rule images produced from the subset of spectral channels were chosen for the final models rather than those produced with all channels as they were ranked as more important. This is consistent with previous results comparing detection accuracies of different channel selections. For example, Cho et al. (2010) used an SAM-based spectral channel selection process to increase overall detection accuracies of savanna tree species. Naidoo et al. (2012) used RF- and SAM-based spectral channel selections and showed that structural information combined with spectral information increases classification accuracy of tree species in RF models. More recent studies utilizing a subset of spectral channels for classification include Somers and Asner (2013) and Kuo et al. (2014), both of which showed an increase in classification accuracies using channel subsets. Additional considerations for the hyperspectral imagery are the phenology of deciduous trees in the study area and the timing of the imagery, as leaf-off conditions for deciduous trees while understory invasive plants have leaves present increases the area for spectral analyses. However, as hyperspectral imagery is often collected to serve multiple purposes, catering the acquisition timing to one or even several species may be difficult. Thus, satellite imagery programs have been well-suited to mapping invasive understory plants in deciduous forests due to their multiple acquisition dates (Bradley, 2014). For example, Resasco et al. (2007), Wilfong et al. (2009), and Shouse et al. (2013) all used Landsat sensors during fall or winter conditions to map the understory invasive plant, honeysuckle (*Lonicera maackii*) across different environments. Kimothi et al. (2010) map *Lantana camara* during leaf-off conditions in India. While these studies successfully mapped plant invasions, the canopies in the forests were dominated by deciduous trees. Such an approach would not cover as wide as breadth in coniferous-dominated forests. However, there is an opportunity to explore utilizing hyperspectral imagery over the deciduous portions of this forest in leaf-off conditions to detect understory invasive plants.

In this present study, LiDAR data effectively modeled the species distributions even without combining it with hyperspectral data, highlighting the applicability of LiDAR data to be a source of information for ecological models. While this has been shown in the past using orographic and structural information derived from LiDAR data, this present study added LiDAR-derived irradiance to the ecological models. In terms of the use of specific LiDAR products, direct irradiance was more highly ranked than diffuse irradiance, hence it being included in the final models. Direct irradiance was also highly ranked for English ivy detection in open areas (**Figure 5**). Consistent with the known habitat preferences of the English ivy for cooler, shaded areas, and of Himalayan blackberry for open areas (Clergeau, 1992; Gaire et al., 2015), Himalayan blackberry preferred areas with more direct irradiance than English ivy (**Figure 8**), indicating that when in open areas, English ivy still established in shaded locations. Direct irradiance was likely important for determining the areas of English ivy in open areas due to the limited sites at which direct irradiance conditions suit English ivy in open areas. Forest structural characteristics were more important than orographic variables in areas with closed canopies for both species (**Figure 5**). Specifically, measures of the vertical distribution of LiDAR points, the coefficient of variation of height and skewness, were important predictors (**Figure 5**). Based on **Figure 8**, English ivy had a different preference for coefficient of variation than absent plots. This indicates that English ivy either established in sites with certain structural characteristics, influences the structure, or both. Previous research indicates that English ivy plants prefer shaded forests with trees with large trunks (Schnitzler and Heuzé, 2006), which in Surrey may be forest stands with relatively tall, older trees of similar ages. Additionally, as English ivy can decrease the amount of understory cover by other species (Biggerstaff and Beck, 2007), many of which in Surrey are taller shrubs; the standard deviation of height of these stands may decrease.

While Himalayan blackberry and English ivy distributions may be more related to forest structural characteristics than orographic ones, the pixel resolution may influence this signal. Gu et al. (2015) used a pixel size of 20 m to map forest composition with LiDAR-derived forest structural variables and hyperspectral imagery, and found that the imagery was more important for their purposes, perhaps due to the limitations caused by the large pixel size. Numerous studies have shown that increasing the coarseness of the spatial resolution of hyperspectral imagery can decrease its applicability for detecting species (e.g., Underwood et al., 2007; Peña et al., 2013), however, few studies have quantified the effects of LiDAR footprint sizes or point densities for ecological mapping of single understory plant species. A review of some relevant studies indicates that point densities of 25 points per m^2^ sampled to 1 m pixels, as are present in this current study, may be at a finer scale than needed for modeling or management purposes as follows. One study focusing on estimating biomass of one understory species from LiDAR data found that pixel sizes between 5 and 30 m produced better models than those at 1 m (Li et al., 2015). As previously mentioned Singh et al. (2015) mapped understory plant invasions at a 5 m pixel size. Barber et al. (2016) used LiDAR data with 1.4 points per m^2^ sampled to 25 m pixels to estimate understory shrub abundance based on ecological relationships. Furthermore, Brubaker et al. (2013) tested the accuracies of LiDAR-derived DEMs and differences between derived products such as curvature, slope, and roughness and found no substantial differences between DEMs produced from LiDAR data with more than 10 points per m^2^ and less than 1 point per m^2^. As evidenced by these varying results, point densities, and pixel sizes, more research is needed to quantify ideal LiDAR resolutions for different applications. Moreover, in urban areas where LiDAR data is often collected for multiple purposes (e.g., engineering, flood control, forestry), the data may need to be downgraded after acquisition to suit certain purposes.

The land cover classification, a product of both the hyperspectral imagery and the LiDAR data, was ranked as the variable with the lowest importance in all models (**Figure 5**). Because water, paved areas, and buildings were masked, only coniferous forests, deciduous forests, and areas of grass were considered in the RF model. The spatial breadth of these classes may have contributed to the low importance of the land cover classification, as other variables could likely better explain the spatial distributions of the invasive species. However, analysis of the locations of the invasions found that Himalayan blackberry and English ivy differed in abundance between deciduous and coniferous forests (**Figure 7**), with Himalayan blackberry showing a preference for deciduous forests and English ivy for coniferous forests, consistent with the habitat preferences of the two species (Clergeau, 1992; Gaire et al., 2015).

Analysis of the distance between roads and invasion showed that both absolute coverage of Himalayan blackberry and English ivy was inversely related to the distance from roads (**Figure 9**). These results have management implications, as they indicate target areas for city managers to direct resources toward curbing the spread of invasion. Additionally, targeting areas near roads may be relatively low effort as field crews can access invasion patches without traveling far into urban natural areas. However, when considered proportionally, the area covered by Himalayan blackberry and English ivy was not related to the distances from roads (**Figure 9**). This contradicts the general trend shown in previous research on English ivy ecology, which shows decreasing proportional cover of English ivy with increasing distance from roads (Arévalo et al., 2008). However, this previous study differs from the present study in that it did not occur in an urban area; the forest was made up of mostly deciduous trees, and only analyzed species composition up to 60 m from roads. The distance to impervious surfaces, while not directly analogous to distance to roads, is also indicative of human activity and is somewhat important for predicting Himalayan blackberry distributions in open areas and areas with closed canopies according to **Figure 5**. **Figure 8** shows that occurrences in open areas are more frequently closer to impervious surface than in areas with closed canopies, but this is likely due to there being more open area closer to impervious areas. One reason that impervious surfaces may not be a strong indicator of invasions in this study are that impervious surfaces relate better to the beginning of invasions, but once invasions are established, the relationship weakens (Beauséjour et al., 2015). Another reason may be that because this study is in an urban area, all areas are close enough to roads and impervious surfaces not enough variation exists to observe a substantial effect. Additionally, both roads and impervious surfaces aid in dispersal, but there may be other variables that ultimately lead to the invasion remaining on the landscape (Beauséjour et al., 2015).

This present study highlights a trend in ecological and remote sensing research of combining the two disciplines. Often remote sensing scientists create data products without consideration for how the products are going to be used. In these cases, ecologists must find ways to cater these products to their research. This present study shows that processing remotely sensed data specifically for ecological modeling of certain plant species is effective. Therefore, land managers and researchers modeling invasive species distributions should consider the goals of the modeling in conjunction with the spectral and spatial resolutions of remotely sensed data before processing it.

## Conclusion

Current municipal operational approaches for detecting plant invasions rely on field crews that cannot produce spatially contiguous information across a large area about the distribution of plant species. Remote sensing technologies can augment this approach by detecting plants across large urban areas with a single methodology. This study produced detection models for Himalayan blackberry and English ivy across Surrey, BBC, Canada. RF models were used in conjunction with variables from hyperspectral imagery and LiDAR data to detect Himalayan blackberry and English ivy invasions across open and forested areas of the city. Spatial relationships on the resulting maps were quantified by analyzing clusters and relationships with environmental variables. The resulting RF detection models classified Himalayan blackberry with 78.8 and 87.8% accuracies and English ivy with 81.9 and 82.1% accuracies, with plants in open areas being better detected than those in areas with closed canopies. Spatial analysis of the resulting maps showed that Himalayan blackberry occurred more often in deciduous forests whereas English ivy tended to occur in coniferous forests. Both species were found to have a negative relationship with distance from roads when considering absolute area. This study highlights the applicability of LiDAR data and hyperspectral imagery in mapping plant species, specifically invasive plant species distributions in open areas and areas with closed canopies. This study also demonstrates that creating LiDAR- and hyperspectral-derived products specific to species to is an effective way to model their distributions. Furthermore, this present study shows that novel LiDAR-derived products, such as irradiance models, may be useful for ecological models over large extents. Some limitations of the study include its reliance on data from public land and its applicability to only mature plants. Future research could address these limitations by broadening the training datasets to include private property and plants of different life stages.

## Author Contributions

CC: Wrote and compiled the manuscript, performed analyses and data processing. NC: Wrote and edited parts of the manuscript. advised on which analyses to perform and how to process to the data. AP: Processed data for the land cover classification section and wrote parts of the results and methods that corresponded to the land cover classifications. TT: Provided code, advice, and edits related to the irradiance models. AC: Provided edits and suggested analyses related to the irradiance models and random forest classification results. NA: Provided data used in this paper and manuscript edits.

## Conflict of Interest Statement

The authors declare that the research was conducted in the absence of any commercial or financial relationships that could be construed as a potential conflict of interest.

## References

[B1] Adam E. Mutanga O. Rugege D. (2010). Multispectral and hyperspectral remote sensing for identification and mapping of wetland vegetation: a review. *Wetl. Ecol. Manag.* 18 281–296. 10.1007/s11273-009-9169-z

[B2] Akbari H. Pomerantz M. Taha H. (2001). Cool surfaces and shade trees to reduce energy use and improve air quality in urban areas. *Sol. Energy* 70 295–310. 10.1016/S0038-092X(00)00089-X

[B3] Allouche O. Tsoar A. Kadmon R. (2006). Assessing the accuracy of species distribution models: prevalence, kappa and the true skill statistic (TSS). *J. Appl. Ecol.* 43 1223–1232. 10.1111/j.1365-2664.2006.01214.x

[B4] Amor R. L. (1973). Ecology and control of blackberry (*Rubus fruticosus* L. agg.). *Weed Res.* 13 218–223. 10.1111/j.1365-3180.1973.tb01266.x

[B5] Andrew M. E. Ustin S. (2008). The role of environmental context in mapping invasive plants with hyperspectral image data. *Remote Sens. Environ.* 112 4301–4317. 10.1016/j.rse.2008.07.016

[B6] Andrew M. E. Ustin S. L. (2009). Habitat suitability modelling of an invasive plant with advanced remote sensing data. *Divers. Distrib.* 15 627–640. 10.1111/j.1472-4642.2009.00568.x

[B7] Anselin L. (1995). Local indicators of spatial association - LISA. *Geogr. Anal.* 27 93–115. 10.1111/j.1538-4632.1995.tb00338.x

[B8] Arévalo J. R. Delgado J. D. Fernández-Palacios J. M. (2008). Changes in plant species composition and litter production in response to roads and trails in the laurel forest of Tenerife (Canary Islands). *Plant Biosyst.* 142 614–622. 10.1080/11263500802410991

[B9] Arnberger A. (2006). Recreation use of urban forests: an inter-area comparison. *Urban For. Urban Green.* 4 135–144. 10.1016/j.ufug.2006.01.004

[B10] Asner G. P. Knapp D. E. Kennedy-Bowdoin T. Jones M. O. Martin R. E. Boardman J. (2008). Invasive species detection in Hawaiian rainforests using airborne imaging spectroscopy and LiDAR. *Remote Sens. Environ.* 112 1942–1955. 10.1016/j.rse.2007.11.016

[B11] Asner G. P. Vitousek P. M. (2005). Remote analysis of biological invasion and biogeochemical change. *Proc. Natl. Acad. Sci. U.S.A.* 102 4383–4386. 10.1073/pnas.0500823102 15761055 PMC554001

[B12] Astley C. (2010). *How Does Himalayan Blackberry (Rubus Armeniacus) Impact Breeding Bird Diversity? A Case Study of the Lower Mainland of British Columbia*. Victoria, BC: Royal Roads University.

[B13] Axelsson P. E. (1999). Processing of laser scanner data - algorithms and applications. *ISPRS J. Photogramm. Remote Sens.* 54 138–147. 10.1016/S0924-2716(99)00008-8

[B14] Bachman C. G. (1979). *Laser Radar Systems and Techniques*. Dedham, MA: Artech House, Inc.

[B15] Barber Q. E. Bater C. W. Braid A. C. R. Coops N. C. Tompalski P. Nielsen S. E. (2016). Airborne laser scanning for modelling understory shrub abundance and productivity. *For. Ecol. Manag.* 377 46–54. 10.1016/j.foreco.2016.06.037

[B16] Barnea A. Harborne J. B. Pannell C. (1993). What parts of fleshy fruits contain secondary compounds toxic to birds and why? *Biochem. Syst. Ecol.* 21 421–429. 10.1016/0305-1978(93)90100-6

[B17] Beauséjour R. Handa I. T. Lechowicz M. J. Gilbert B. Vellend M. (2015). Historical anthropogenic disturbances influence patterns of non-native earthworm and plant invasions in a temperate primary forest. *Biol. Invasions* 17 1267–1281. 10.1007/s10530-014-0794-y

[B18] Bentivegna D. J. Smeda R. J. Wang C. (2012). Detecting cutleaf teasel (*Dipsacus laciniatus*) along a Missouri highway with hyperspectral imagery. *Invasive Plant Sci. Manag.* 5 155–163. 10.1614/IPSM-D-10-00053.1

[B19] Beven K. J. Kirkby M. J. (1979). A physically based, variable contributing area model of basin hydrology. *Hydrol. Sci. Bull.* 24 43–69. 10.1080/02626667909491834

[B20] Biggerstaff M. S. Beck W. (2007). Effects of method of English Ivy removal and seed addition on regeneration of vegetation in a southeastern piedmont forest. *Am. Midl. Nat.* 158 206–220. 10.1674/0003-0031(2007)158[206:EOMOEI]2.0.CO;2

[B21] Bradley B. A. (2014). Remote detection of invasive plants: a review of spectral, textural and phenological approaches. *Biol. Invasions* 16 1411–1425. 10.1007/s10530-013-0578-9

[B22] Breiman L. (2001). Random forests. *Mach. Learn.* 45 5–32. 10.1023/A:1017934522171

[B23] Brubaker K. M. Myers W. L. Drohan P. J. Miller D. A. Boyer E. W. (2013). The use of LiDAR terrain data in characterizing surface roughness and microtopography. *Appl. Environ. Soil Sci.* 2013:891534 10.1155/2013/891534

[B24] Calviño-Cancela M. Méndez-Rial R. Reguera-Salgado J. Martín-Herrero J. (2014). Alien plant monitoring with ultralight airborne imaging spectroscopy. *PLoS ONE* 9:e102381 10.1371/journal.pone.0102381 PMC4092129 25010601

[B25] Caplan J. S. Yeakley J. A. (2006). Rubus armeniacus (Himalayan blackberry) occurrence and growth in relation to soil and light conditions in western Oregon. *Northwest Sci.* 80 9–17.

[B26] Chance C. M. Coops N. C. Crosby K. Aven N. (2016). Spectral wavelength selection and detection of two invasive plant species in an urban area. *Can. J. Remote Sens.* 42 1–14. 10.1080/07038992.2016.1143330

[B27] Cho M. A. Debba P. Mathieu R. Naidoo L. Van Aardt J. Asner G. P. (2010). Improving discrimination of savanna tree species through a multiple-endmember spectral angle mapper approach: canopy-level analysis. *IEEE Trans. Geosci. Remote Sens.* 48 4133–4142.

[B28] Clark M. Roberts D. Clark D. (2005). Hyperspectral discrimination of tropical rain forest tree species at leaf to crown scales. *Remote Sens. Environ.* 96 375–398. 10.1016/j.rse.2005.03.009

[B29] Clergeau P. (1992). The effects of birds on seed germination of fleshy-fruit plants in termperate farmland. *Acta Oecol.* 13 679–686.

[B30] Coops N. C. Hilker T. Wulder M. A. St-Onge B. Newnham G. J. Siggins A. (2007). Estimating canopy structure of Douglas-fir forest stands from discrete-return LiDAR. *Trees Struct. Funct.* 21 295–310. 10.1007/s00468-006-0119-6

[B31] Curran P. J. (1989). Remote sensing of foliar chemistry. *Remote Sens. Environ.* 30 271–278. 10.1016/0034-4257(89)90069-2

[B32] Cutler D. R. Edwards T. C. Beard K. H. Cutler A. Hess K. T. Gibson J. (2007). Random forests for classification in ecology. *Ecology* 88 2783–2792. 10.1890/07-0539.1 18051647

[B33] Datt B. (1998). Remote sensing of chlorophyll a, chlorophyll b, chlorophyll a+b, and total carotenoid content in eucalyptus leaves. *Remote Sens. Environ.* 66 111–121. 10.1016/S0034-4257(98)00046-7

[B34] Dlugosch K. M. (2005). Understory community changes associated ith English Ivy invasions in Seattle’s urban parks. *Northwest Sci.* 79 52–59.

[B35] Ehrenfeld J. G. (2003). Effects of exotic plant invasions on soil nutrient cycling processes. *Ecosystems* 6 503–523. 10.1007/s10021-002-0151-3

[B36] Ellstrand N. C. Schierenbeck K. A. (2000). Hybridization as a stimulus for the evolution of invasiveness in plants? *Proc. Natl. Acad. Sci.* 97 7043–7050. 10.1073/pnas.97.13.7043 10860969 PMC34382

[B37] Escobedo F. J. Kroeger T. Wagner J. E. (2011). Urban forests and pollution mitigation: analyzing ecosystem services and disservices. *Environ. Pollut.* 159 2078–2087. 10.1016/j.envpol.2011.01.010 21316130

[B38] Fernández-Delgado M. Cernadas E. Barro S. Amorim D. (2014). Do we need hundreds of classifers to solve real world classification problems. *J. Mach. Learn. Res.* 15 3133–3181.

[B39] Fierke M. K. Kauffman J. B. (2006). Invasive species influence riparian plant diversity along a successional gradient, Willamette River, Oregon. *Nat. Areas J.* 26 376–382. 10.3375/0885-8608(2006)26[376:ISIRPD]2.0.CO;2

[B40] Fu W. J. Jiang P. K. Zhou G. M. Zhao K. L. (2014). Using Moran’s I and GIS to study the spatial pattern of forest litter carbon density in a subtropical region of southeastern China. *Biogeosciences* 11 2401–2409. 10.5194/bg-11-2401-2014

[B41] Gaire R. Astley C. Upadhyaya M. K. Clements D. R. Bargen M. (2015). The Biology of Canadian Weeds. 154. Himalayan blackberry. *Can. J. Plant Sci.* 95 557–570. 10.4141/cjps-2014-402

[B42] Grena R. (2008). An algorithm for the computation of the solar position. *Sol. Energy* 82 462–470. 10.1016/j.solener.2007.10.001

[B43] Große-Stoltenberg A. Werner C. Hellmann C. Oleland J. Thiele J. (2016). Evaluation of continuous VNIR-SWIR spectra versus narrowband hyperspectral indices to discriminate the invasive Acacia longifolia within a Mediterranean dune ecosystem. *Remote Sens.* 8:334 10.3390/rs8040334

[B44] Gu H. Singh A. Townsend P. A. (2015). Detection of gradients of forest composition in an urban area using imaging spectroscopy. *Remote Sens. Environ.* 167 168–180. 10.1016/j.rse.2015.06.010

[B45] Gundale M. J. Sutherland S. Deluca T. H. (2008). Fire, native species, and soil resource interactions influence the spatio-temporal invasion pattern of *Bromus tectorum*. *Ecography* 31 201–210. 10.1111/j.0906-7590.2008.5303.x

[B46] Hammer A. Heinemann D. Hoyer C. Kuhlemann R. Lorenz E. Müller R. (2003). Solar energy assessment using remote sensing technologies. *Remote Sens. Environ.* 86 423–432. 10.1016/S0034-4257(03)00083-X

[B47] Harmer R. Peterken G. Kerr G. Poulton P. (2001). Vegetation changes during 100 years of development of two secondary woodlands on abandoned arable land. *Biol. Conserv.* 101 291–304. 10.1016/S0006-3207(01)00072-6

[B48] Hartman K. M. McCarthy B. C. (2008). Changes in forest structure and species composition following invasion by a non-indigenous shrub, Amur Honeysuckle (*Lonicera maackii*). *J. Torrey Bot. Soc.* 135 245–259. 10.3159/07-RA-036.1

[B49] Hawthorne T. L. Elmore V. Strong A. Bennett-Martin P. Finnie J. Parkman J. (2015). Mapping non-native invasive species and accessibility in an urban forest: a case study of participatory mapping and citizen science in Atlanta, Georgia. *Appl. Geogr.* 56 187–198. 10.1016/j.apgeog.2014.10.005

[B50] He K. S. Rocchini D. Neteler M. Nagendra H. (2011). Benefits of hyperspectral remote sensing for tracking plant invasions. *Divers. Distrib.* 17 381–392. 10.1111/j.1472-4642.2011.00761.x

[B51] Hofierka J. Suri M. Šúri M. (2002). “The solar radiation model for Open source GIS: implementation and applications,” in *Proceedings of the Open Source GIS-GRASS Users Conference*, Trento, Italy.

[B52] Hough R. L. (2014). Biodiversity and human health: evidence for causality? *Biodivers. Conserv.* 23 267–288. 10.1007/s10531-013-0614-1

[B53] Huang C.-Y. Asner G. P. (2009). Applications of remote sensing to alien invasive plant studies. *Sensors* 9 4869–4889. 10.3390/s90604869 22408558 PMC3291943

[B54] Ishii J. Washitani I. (2013). Early detection of the invasive alien plant Solidago altissima in moist tall grassland using hyperspectral imagery. *Int. J. Remote Sens.* 34 5926–5936. 10.1080/01431161.2013.799790

[B55] Jansen F. Ewald J. Zerbe S. (2011). Ecological preferences of alien plant species in North-Eastern Germany. *Biol. Invasions* 13 2691–2701. 10.1007/s10530-011-9939-4

[B56] Jones J. M. White I. R. White J. M. L. McFadden J. P. (2009). Allergic contact dermatitis to English ivy (*Hedera helix*) – a case series. *Contact Dermat.* 60 179–180. 10.1111/j.1600-0536.2008.01492.x 19260922

[B57] Jones T. G. Coops N. C. Sharma T. (2010). Employing ground-based spectroscopy for tree-species differentiation in the Gulf Islands National Park Reserve. *Int. J. Remote Sens.* 31 1121–1127. 10.1080/01431160903349040

[B58] Kashani A. G. Olsen M. J. Parrish C. E. Wilson N. (2015). A review of LIDAR radiometric processing: from ad hoc intensity correction to rigorous radiometric calibration. *Sensors* 15 28099–28128. 10.3390/s151128099 26561813 PMC4701271

[B59] Kim S. McGaughey R. J. Andersen H.-E. Schreuder G. (2009). Tree species differentiation using intensity data derived from leaf-on and leaf-off airborne laser scanner data. *Remote Sens. Environ.* 113 1575–1586. 10.1016/j.rse.2009.03.017

[B60] Kimothi M. M. Anitha D. Vasistha H. B. Soni P. Chandola S. K. (2010). Remote sensing to map the invasive weed, Lantana camara in forests. *Trop. Ecol.* 51 67–74.

[B61] Kowarik I. (1995). “Time lags in biological invasions with regard to the success and failure of alien species,” in *Plant Invasions: General Aspects and Special Problems*, eds Pysek P. Prach K. Rejmanek M. Wade M. (Amsterdam: SPB Academic Publishing), 15–38.

[B62] Kraus K. Pfeifer N. (1998). Determination of terrain models in wooded areas with airborne laser scanner data. *ISPRS J. Photogramm. Remote Sens.* 53 193–203. 10.1016/S0924-2716(98)00009-4

[B63] Kruse F. A. Lefkoff A. B. Boardman J. B. Heidebrecht K. B. Shapiro A. T. Barloon P. J. (1993). The spectral image processing system (SIPS) - interactive visualization and analysis of imaging spectrometer data. *Remote Sens. Environ.* 44 145–163. 10.1016/0034-4257(93)90013-N

[B64] Kuebbing S. E. Classen A. T. Simberloff D. (2014). Two co-occurring invasive woody shrubs alter soil properties and promote subdominant invasive species. *J. Appl. Ecol.* 51 124–133. 10.1111/1365-2664.12161

[B65] Kuo B. C. Ho H. H. Li C. H. Hung C. C. Taur J. S. (2014). A kernel-based feature selection method for SVM with RBF kernel for hyperspectral image classification. *IEEE J. Sel. Top. Appl. Earth Obs. Remote Sens.* 7 317–326. 10.1109/JSTARS.2013.2262926

[B66] Kuo F. E. (2003). Social Aspects of Urban Forestry: the role of arboriculture in a healthy social ecology. *J. Aboric.* 29 148–155.

[B67] Laaidi M. Laaidi K. Besancenot J. P. Thibaudon M. (2003). Ragweed in France: an invasive plant and its allergenic pollen. *Ann. Allergy Asthma Immunol.* 91 195–201. 10.1016/S1081-1206(10)62177-1 12952115

[B68] Lampinen J. Ruokolainen K. Huhta A.-P. (2015). Urban power line corridors as novel habitats for grassland and alien plant species in South-Western Finland. *PLoS ONE* 10:e0142236 10.1371/journal.pone.0142236 PMC4643934 26565700

[B69] Lawrence R. L. Wood S. D. Sheley R. L. (2006). Mapping invasive plants using hyperspectral imagery and Breiman Cutler classifications (randomForest). *Remote Sens. Environ.* 100 356–362. 10.1016/j.rse.2005.10.014

[B70] Li A. Glenn N. F. Olsoy P. J. Mitchell J. J. Shrestha R. (2015). Aboveground biomass estimates of sagebrush using terrestrial and airborne LiDAR data in a dryland ecosystem. *Agric. For. Meteorol.* 213 138–147. 10.1016/j.agrformet.2015.06.005

[B71] Liaw A. Wiener M. (2002). Classification and Regression by randomForest. *R News* 2 18–22.

[B72] Loosvelt L. Peters J. Skriver H. Lievens H. Van Coillie F. M. B. De Baets B. (2012). Random Forests as a tool for estimating uncertainty at pixel-level in SAR image classification. *Int. J. Appl. Earth Obs. Geoinf.* 19 173–184. 10.1016/j.jag.2012.05.011

[B73] Mack R. N. Simberloff D. Lonsdale W. M. Evans H. Clout M. Bazzaz F. A. (2000). Biotic invasions: causes, epidemiology, global consequences, and control. *Ecol. Appl.* 10 689–710. 10.1890/1051-0761(2000)010[0689:BICEGC]2.0.CO;2

[B74] Mcpherson G. Simpson J. R. Peper P. J. Maco S. E. Xiao Q. (2005). Municipal forest benefits and costs in five US Cities. *J. For.* 103 411–416.

[B75] Metcalfe D. J. (2005). Hedera helix L. *J. Ecol.* 93 632–648. 10.1111/j.1365-2745.2005.01021.x

[B76] Millard K. Richardson M. (2015). On the importance of training data sample selection in Random Forest image classification: a case study in peatland ecosystem mapping. *Remote Sens.* 7 8489–8515. 10.3390/rs70708489

[B77] Mirik M. Ansley R. J. Steddom K. Jones D. Rush C. Michels G. (2013). Remote distinction of a noxious weed (Musk Thistle: Carduus Nutans) using airborne hyperspectral imagery and the support vector machine classifier. *Remote Sens.* 5 612–630. 10.3390/rs5020612

[B78] Moran P. (1950). Notes on continuous stochastic phenomena. *Biometrika* 37 17–23. 10.2307/2332142 15420245

[B79] Mutanga O. Skidmore A. K. Prins H. H. T. (2004). Predicting in situ pasture quality in the Kruger National Park, South Africa, using continuum-removed absorption features. *Remote Sens. Environ.* 89 393–408. 10.1016/j.rse.2003.11.001

[B80] Naidoo L. Cho M. A. Mathieu R. Asner G. (2012). Classification of savanna tree species, in the Greater Kruger National Park region, by integrating hyperspectral and LiDAR data in a Random Forest data mining environment. *ISPRS J. Photogramm. Remote Sens.* 69 167–179. 10.1016/j.isprsjprs.2012.03.005

[B81] Narumalani S. Mishra D. R. Wilson R. Reece P. Kohler A. (2009). Detecting and mapping four invasive species along the floodplain of North Platte River, Nebraska. *Weed Technol.* 23 99–107. 10.1614/WT-08-007.1

[B82] Ørka H. O. Næsset E. Bollandsås O. M. (2009). Classifying species of individual trees by intensity and structure features derived from airborne laser scanner data. *Remote Sens. Environ.* 113 1163–1174. 10.1016/j.rse.2009.02.002

[B83] Parendes L. A. Jones J. A. (2000). Role of Light Availability and Dispersal in Exotic Plant Invasion along Roads and Streams in the H. J. Andrews Experimental Forest, Oregon. *Conserv. Biol.* 14 64–75.

[B84] Parker Williams A. Hunt E. R. (2002). Estimation of leafy spurge cover from hyperspectral imagery using mixture tuned matched filtering. *Remote Sens. Environ.* 82 446–456. 10.1016/S0034-4257(02)00061-5

[B85] Paulsen E. Christensen L. P. Andersen K. E. (2010). Dermatitis from common ivy (*Hedera helix* L. subsp. *helix*) in Europe: past, present, and future. *Contact Dermat.* 62 201–209. 10.1111/j.1600-0536.2009.01677.x 20236156

[B86] Pearson R. G. Dawson T. P. Liu C. (2004). Modelling species distributions in Britain: a hierarchical integration of climate and land-cover data. *Ecography* 27 285–298. 10.1111/j.0906-7590.2004.03740.x

[B87] Pejchar L. Mooney H. A. (2009). Invasive species, ecosystem services and human well-being. *Trends Ecol. Evol.* 24 497–504. 10.1016/j.tree.2009.03.016 19577817

[B88] Peña M. A. Cruz P. Roig M. (2013). The effect of spectral and spatial degradation of hyperspectral imagery for the Sclerophyll tree species classification. *Int. J. Remote Sens.* 34 7113–7130. 10.1080/01431161.2013.817712

[B89] Pimentel D. Lach L. Zuniga R. Morrison D. (2000). Environmental and economic costs of nonindigenous species in the United States. *Bioscience* 50 53–65. 10.1641/0006-3568(2000)050[0053:EAECON]2.3.CO;2

[B90] Pimentel D. Zuniga R. Morrison D. (2005). Update on the environmental and economic costs associated with alien-invasive species in the United States. *Ecol. Econ.* 52 273–288. 10.1016/j.ecolecon.2004.07.013

[B91] Price C. (2003). Quantifying the aesthetic benefits of urban forestry. *Urban For. Urban Green.* 1 123–133. 10.1078/1618-8667-00013

[B92] Pysek P. (1998). Alien and native species in Central European urban floras: a quantitative comparison. *J. Biogeogr.* 25 155–163. 10.1046/j.1365-2699.1998.251177.x

[B93] Pysek P. Hulme P. E. (2005). Spatio-temporal dynamics of plant invasions: linking pattern to process. *Ecoscience* 12 302–315. 10.2980/i1195-6860-12-3-302.1

[B94] Resasco J. Hale A. N. Henry M. C. Gorchov D. L. (2007). Detecting an invasive shrub in a deciduous forest understory using late fall Landsat sensor imagery. *Int. J. Remote Sens.* 28 3739–3745. 10.1080/01431160701373721

[B95] Richter R. Schlapfer D. (2016). *ATC* *OR-4 User Guide* Available at: http://www.rese.ch/pdf/atcor4_manual.pdf

[B96] Rocchini D. Andreo V. Forster M. Garzon-Lopez C. X. Gutierrez A. P. Gillespie T. W. (2015). Potential of remote sensing to predict species invasions: a modelling perspective. *Progr. Phys. Geogr.* 39 283–309. 10.1177/0309133315574659

[B97] Roth K. L. Roberts D. A. Dennison P. E. Peterson S. H. Alonzo M. (2015). The impact of spatial resolution on the classification of plant species and functional types within imaging spectrometer data. *Remote Sens. Environ.* 171 45–57. 10.1016/j.rse.2015.10.004

[B98] Roy D. B. Hill M. Rothery P. Effects P. (1999). Effects of urban land cover on the local species pool in Britain. *Ecography* 22 507–515. 10.1111/j.1600-0587.1999.tb01279.x

[B99] Royo A. A. Carson W. P. (2006). On the formation of dense understory layers in forests worldwide: consequences and implications for forest dynamics, biodiversity, and succession. *Can. J. For. Res.* 36 1345–1362. 10.1139/x06-025

[B100] Schnitzler A. Heuzé P. (2006). Ivy (*Hedera helix* L.) dynamics in riverine forests: effects of river regulation and forest disturbance. *For. Ecol. Manag.* 236 12–17. 10.1016/j.foreco.2006.05.060

[B101] Schwartz M. W. Porter D. J. Randall J. M. Lyons K. E. (1996). “Impact of nonindigenous plants,” in *Proceedings of the Sierra Nevada Ecosystem Project: Final Report to Congress, Assessments and Scientific Basis for Management Options*, Vol. 2 (Davis, CA: University of California).

[B102] Shaw G. A. Burke H. K. (2003). Spectral imaging for remote sensing. *Lincoln Lab. J.* 14 3–28.

[B103] Shouse M. Liang L. Fei S. (2013). Identification of understory invasive exotic plants with remote sensing in urban forests. *Int. J. Appl. Earth Obs. Geoinf.* 21 525–534. 10.1016/j.jag.2012.07.010

[B104] Singh K. K. Davis A. J. Meentemeyer R. K. (2015). Detecting understory plant invasion in urban forests using LiDAR. *Int. J. Appl. Earth Obs. Geoinf.* 38 267–279. 10.1016/j.jag.2015.01.012

[B105] Somers B. Asner G. P. (2013). Multi-temporal hyperspectral mixture analysis and feature selection for invasive species mapping in rainforests. *Remote Sens. Environ.* 136 14–27. 10.1016/j.rse.2013.04.006

[B106] Somers B. Delalieux S. Verstraeten W. W. van Aardt J. A. N. Albrigo G. L. Coppin P. (2010). An automated waveband selection technique for optimized hyperspectral mixture analysis. *Int. J. Remote Sens.* 31 5549–5568. 10.1080/01431160903311305

[B107] Sørensen R. Zinko U. Seibert J. (2006). On the calculation of the topographic wetness index: evaluation of different methods based on field observations. *Hydrol. Earth Syst. Sci.* 10 101–112. 10.5194/hess-10-101-2006

[B108] Swetnam T. L. Lynch A. M. Falk D. A. Yool S. R. (2015). Discriminating disturbance from natural variation with LiDAR in semi-arid forests in the southwestern USA. *Ecosphere* 6 1–22. 10.1890/ES14-00384.1

[B109] Thomas L. K. (1980). *The Impact of Three Exotic Plant Species on a Potomac Island*. U.S. National Park Service Scientific Monograph Series No 13. Washington DC: Government Printing Office.

[B110] Tooke T. R. Coops N. C. Christen A. Gurtuna O. Prévot A. (2012). Integrated irradiance modelling in the urban environment based on remotely sensed data. *Sol. Energy* 86 2923–2934. 10.1016/j.solener.2012.06.026

[B111] Underwood E. Ustin S. L. DiPietro D. (2003). Mapping nonnative plants using hyperspectral imagery. *Remote Sens. Environ.* 86 150–161. 10.1016/S0034-4257(03)00096-8

[B112] Underwood E. C. Mulitsch M. J. Greenberg J. A. Whiting M. L. Ustin S. L. Kefauver S. C. (2006). Mapping invasive aquatic vegetation in the Sacramento-San Joaquin Delta using hyperspectral imagery. *Environ. Monit. Assess.* 121 47–64. 10.1007/s10661-005-9106-4 16741793

[B113] Underwood E. C. Ustin S. L. Ramirez C. M. (2007). A comparison of spatial and spectral image resolution for mapping invasive plants in coastal california. *Environ. Manag.* 39 63–83. 10.1007/s00267-005-0228-9 17136630

[B114] Valladares F. (2003). Light heterogeneity and plants: from ecophysiology to species coexistence and biodiversity. *Progress Bot.* 64 439–471. 10.1007/978-3-642-55819-1_17

[B115] Wilcove D. S. Rothstein D. Dubow J. Phillips A. Losos E. (1998). Quantifying threats to imperiled species in the United States. *Bioscience* 48 607–615. 10.2307/1313420

[B116] Wilfong B. N. Gorchov D. L. Henry M. C. (2009). Detecting an invasive shrub in deciduous forest understories using remote sensing. *Weed Sci.* 57 512–520. 10.1614/WS-09-012.1

[B117] Wing B. M. Ritchie M. W. Boston K. Cohen W. B. Gitelman A. Olsen M. J. (2012). Prediction of understory vegetation cover with airborne lidar in an interior ponderosa pine forest. *Remote Sens. Environ.* 124 730–741. 10.1016/j.rse.2012.06.024

